# The Immunodominant T-Cell Epitopes of the Mycolyl-Transferases of the Antigen 85 Complex of *M. tuberculosis*

**DOI:** 10.3389/fimmu.2014.00321

**Published:** 2014-07-09

**Authors:** Kris Huygen

**Affiliations:** ^1^Service Immunology, O.D. Communicable and Infectious Diseases, Scientific Institute of Public Health (WIV-ISP), Brussels, Belgium

**Keywords:** antigen 85, mycolyl transferase, Th1 helper T-cell, immunodominance, promiscuous epitopes

## Abstract

The Ag85 complex is a 30–32 kDa family of three proteins (Ag85A, Ag85B, and Ag85C), which all three possess enzymatic mycolyl-transferase activity involved in the coupling of mycolic acids to the arabinogalactan of the cell wall and in the biogenesis of cord factor. By virtue of their strong potential to induce Th1-type immune responses, important for the control of intracellular infections, members of the Ag85 family rank among the most promising TB vaccine candidate antigens. Ag85A and Ag85B, initially purified from *Mycobacterium bovis* bacillus Calmette–Guérin (BCG)/*Mycobacterium tuberculosis* culture filtrate respectively, induce strong T-cell proliferation and IFN-γ production in most healthy individuals latently infected with *M. tuberculosis* and in BCG-vaccinated mice and humans but not in tuberculosis patients. Members of the Ag85 complex are highly conserved in other mycobacterial species. Mice and humans infected with *Mycobacterium ulcerans* or cattle infected with *M. bovis* or *Mycobacterium avium* subsp. *paratuberculosis* also show strong T-cell responses to this protein family. Using synthetic overlapping peptides, bio-informatic prediction programs and tetramer-binding studies, a number of immunodominant CD4^+^ and CD8^+^ T-cell epitopes have been identified in experimental animal models as well as in humans, using proliferation and Th1 cytokine secretion as main read-outs. The results from these studies are summarized in this review.

## Introduction

The Ag85 complex is actually a 30–32 kDa family of three proteins (Ag85A, Ag85B, and Ag85C), which each possess enzymatic mycolyl-transferase activity involved in the coupling of mycolic acids to the arabinogalactan of the cell wall and in the biogenesis of cord factor (1). These proteins are also known for their capacity to bind to the extracellular matrix proteins fibronectin and elastin (2, 3). In literature members of the Ag85 complex are known under different names: ***Mtb*Ag85A**: Rv3804c, P32, FbpA; ***Mtb*** **85B**: Rv1886c, 30 kDa antigen, α-antigen, FbpB; ***Mtb*85C**: Rv0129c, FbpC2; ***M. bovis*85A**: Mb3834c, MPB44; ***M. bovis*** *Ag85B:*MPB59; ***M. bovis*** bacillus Calmette–Guérin **(BCG) 85A**: BCG_3866c; ***M. ulcerans*** **85A**: MUL4987; ***Map*85A**: MAP0216; ***Map*85B**: MAP 1609c.

Members of the Ag85 family are found in all mycobacteria, and sequence comparisons indicate that the Ag85 gene family arose by duplication of an ancestral gene, before the emergence of the actually known mycobacterial species (4). The genes encoding these proteins are not physically linked, but located at distinct sites on the mycobacterial genome. The genes of Ag85 encode for a characteristic leader sequence of about 40 aa, which is cleaved off during export and release of the mature proteins into mycobacterial culture filtrate (CF). The Ag85A and Ag85B components are detected essentially as secreted proteins, whereas the Ag85C component is more tightly associated with the bacterial cell wall envelope. The calculated secretion index of the three proteins reflects this difference in localization (5). The mycolyl-transferase activity of these proteins generates trehalose dimycolate (TDM), an envelope lipid essential for *Mtb* virulence, and cell wall arabinogalactan-linked mycolic acids. A novel inhibitor of Ag85C, 2-amino-6-propyl-4,5,6,7-tetrahydro-1-benzothiophene-3-carbonitrile (I3-AG85) inhibits *Mtb* survival in infected primary macrophages and quantification of mycolic acid-linked lipids of the *Mtb* envelope showed a specific blockade of TDM synthesis (6). Members of the Ag85 complex are highly conserved in other mycobacterial species and mice infected with *Mycobacterium ulcerans* or with some non-tuberculous mycobacteria belonging to the MAIS-group (*Mycobacterium avium, M. intracellulaire*, and *M. scrofulaceum*) show cross-reactive Th1-type immune responses to Ag85 components purified from BCG CF or produced as recombinant *E. coli* derived proteins (5, 7). By virtue of their strong Th1-type cytokine inducing potential, members of the tuberculosis Ag85 complex (particularly the Ag85A and Ag85B component) are among the most promising tuberculosis vaccine candidates today. Many of the new TB vaccines tested in preclinical and clinical trials, are composed of Ag85 components, expressed as recombinant fusion proteins or encoded by recombinant viral vectors (8, 9).

### Amino-acid sequence alignments of Ag85A, Ag85B, Ag85C of *Mtb*, and of Ag85A of *M. ulcerans, M. avium* subsp. *paratuberculosis (Map)*, and *M. leprae*

**Figure 1:**
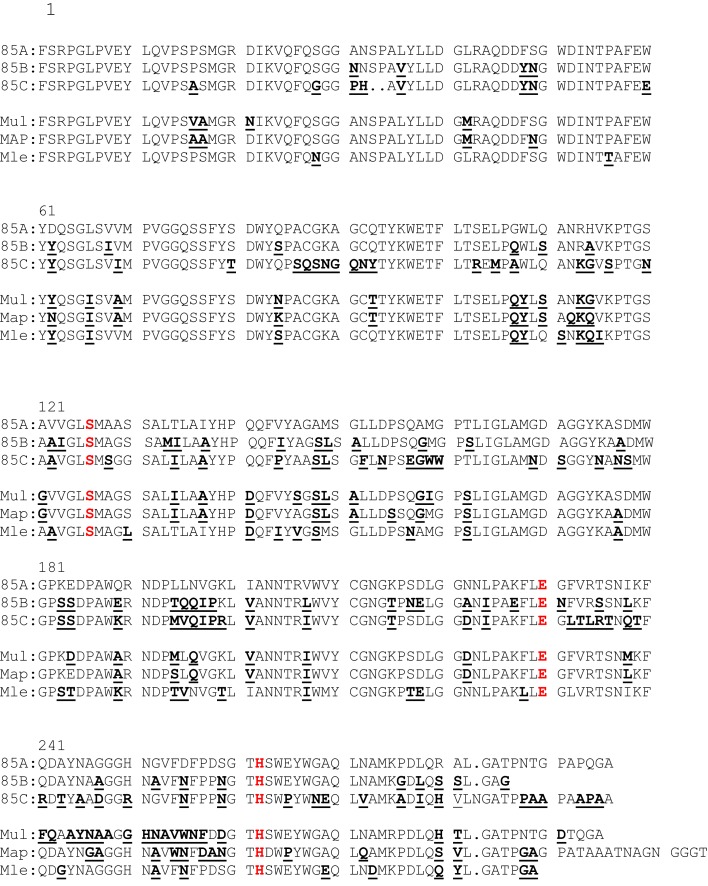
**Amino-acid sequence alignment of *Mtb*Ag85A, *Mtb*Ag85B, *Mtb*Ag85C, and Ag85A sequence of *M. ulcerans, M. avium* subsp. *paratuberculosis*, and *M. leprae***. Aa differences with the *Mtb*Ag85A sequence are underlined and bold. The three aa essential for the mycolyl-transferase activity are indicated in red.

As shown in Figure 1, amino-acid sequences (aa) of the mature Ag85A homologs (without their leader sequence) are highly conserved between mycobacterial species. The three aa essential for the mycolyl-transferase activity, i.e., Serine in position 125, Glutamic acid in position 230 and Histidine in position 262 are conserved in all sequences (highlighted in red). Although some aa stretches are 100% conserved between the different species, there are small variations (indicated in bold as compared to the *Mtb* Ag85A sequence). The aa sequence of Ag85A of *Mycobacterium bovis* and *M. bovis* BCG (1173P2 strain) is identical to the aa sequence of the Ag85A component of *Mtb (*H37Rv) and is therefore not shown. The Ag85B sequence of *M. bovis* differs in one aa from the Ag85B sequence of *Mtb*: Phe100Leu. On the other hand, expression levels of these proteins may differ and whereas Ag85A is the major component in CF of surface-pellicle grown BCG, Ag85B is the major component in CF from *Mtb* and *Map*. The Ag85C component is found in lesser concentrations in the CF, as it is localized more internally in the cell wall. In 2000, M. Horwitz reported that recombinant BCG vaccines expressing the *Mtb* 30-kDa (Ag85B) major secretory protein induced greater protective immunity against tuberculosis than conventional BCG vaccines in a highly susceptible animal model, i.e., the guinea pig (10). The rationale for the construction of this recombinant BCG, was among others the sequence difference of Ag85B of *Mtb* and that of the *M. bovis* BCG Tokyo strain (three aa differences Phe100Leu, Asn245Lys, and Ala246Pro) (11). As already mentioned, Ag85B of other BCG strains and *M. bovis* only differ in position 100 from the sequence of Ag85B of *Mtb*.

### T-cell epitopes of Ag85 of *M. tuberculosis*/*M. bovis* and *M. bovis* BCG (Table 1)

#### Murine studies

##### *M. bovis* BCG

The first T-cell epitope mapping of Ag85A was performed 20 years ago in seven different mouse strains vaccinated with live *M. bovis* BCG (12). Twenty-eight overlapping 20-mer peptides covering the complete mature 295-amino-acid (AA) protein were synthesized. Significant interleukin-2 (IL-2) and gamma interferon (IFN-γ) secretion was measured following *in vitro* stimulation of spleen cells with these peptides. H-2^d^ haplotype mice (BALB/c and DBA/2) reacted preferentially against the amino-terminal half of the protein, i.e., against peptide 5 (aa 41–60) and especially against peptide 11 (aa **101–120**), which contains a predicted I-E^d^ binding motif. H-2^b^ haplotype mice, on the other hand, reacted against peptides from both amino- and carboxy-terminal halves of the protein, peptide 25 (aa **241–260**) and peptide 27 (aa **261–280**) being the most potent stimulators of IL-2 and IFN-γ production. Finally, CBA/J (H-2^k^) and major histocompatibility complex class II mutant B6.C.bml2 mice, carrying a mutant I-A^bml2^ allele on an H-2^b^ background, reacted only very weakly to the 85A peptides. (12).

**Table 1:** **Summary of immunodominant Ag85 T-cell epitopes of *M. tuberculosis***.

Infection/vaccination	Position	Sequence	Restriction	Host	Reference
***M. tuberculosis* INFECTION**					
Rv3804c	241–260	QDAYNAGGGH NGVFDFPDSG	**I-A^b^**	**Mouse**	(18)
Rv1886c	240–254	**F**QDAYNA**A**GGHN**A**VF	**I-A^b^**	**Mouse**	(14)
Rv3804c	101–120	LTSELPGWLQANRHVKPTGS	**I-E^d^**	**Mouse**	(18)
Rv3804c	151–170	GLLDPSQAMG PTLIGLAMGD	**H-2^d^**	**Mouse**	(13)
Rv3804c	191–210	NDPLLNVGKL IANNTRVWVY	**H-2^d^**	**Mouse**	(13)
Rv3804c	51–70	WDINTPAFEWYDQSGLSVVM	**Promiscuous**	**LTBI**	(23)
Rv3804c	141–160	QQFVYAGAMSGLLDPSQAMG	**Promiscuous**	**LTBI**	(23)
Rv1886c	100–117	**F**LTSELP**Q**WL**S**ANR**A**VKP	**Promiscuous**	**LTBI**	(24, 25)
Rv1886c	91–115	GCQTYKWETFLTSEL	**Promiscuous**	**LTBI**	(26)
Rv1886c	193–214	P **TQQIP**KL**V**ANNTR**L**WVYCGNG	**Promiscuous**	**LTBI**	(26)
Rv0129c	70–79	MPVGGQSSFY	HLA-B*35	**Human/*in silico***	(29)
Rv0129c	160–168	WPTLIGLAM	HLA-B*35	**Human/*in silico***	(29)
Rv1886c	224–232	IPA**E**FL 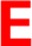 NF	HLA-B*35	**Human/*in silico***	(29)
Rv3804c/Rv1886c	90–104	**A**GCQTYKWETFLTSE	DPB1*04:01	**LTBI**	(28)
***M. bovis* BCG VACCINATION**					
Rv3804c	101–120	LTSELPGWLQANRHVKPTGS	**I-E^d^**	**Mouse**	(12)
Rv3804c	241–260	QDAYNAGGGH NGVFDFPDSG	**I-A^b^**	**Mouse**	(12)
Rv3804c	261–280	T 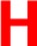 SWEYWGAQ LNAMKPDLQR	**I-A^b^**	**Mouse**	(12)
MPB59	51–70	WDINTPAFEW Y**Y**QSGLS**I**VM	**Promiscuous**	**Human**	(32)
MPB59	11–30	LQVPSPSMGR DIKVQFQSGG	**Promiscuous**	**Human**	(32)
***Mtb* PLASMID DNA VACCINATION**					
Rv3804c	101–120	LTSELPGWLQANRHVKPTGS	**I-E^d^**	**Mouse**	(13, 18)
Rv1886c	100–117	**F**LTSELP**Q**WL**S**ANR**A**VKP	**I-A^d^**	**Mouse**	(13)
Rv0129c	101–120	LT**R**E**M**P**A**WLQAN**KG**V**S**PTG**N**	**H-2^d^**	**Mouse**	(13)
Rv3804c/Rv1886c	141–160	QQFV**Y**AGAMSG**L**LDPSQAMG	**H-2^d^**	**Mouse**	(18)
Rv3804c	191–120	NDPLLNVGKLIANNTRVWVY	**H-2^d^**	**Mouse**	(13)
Rv0129c	191–210	NDP**MVQIPR**L**V**ANNTR**I**WVY	**H-2^d^**	**Mouse**	(13)
Rv3804c	241–260	QDAYNAGGGH NGVFDFPDSG	**I-A^b^**	**Mouse**	(13)
Rv3804c	261–280	T 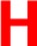 SWEYWGAQ LNAMKPDLQR	**I-A^b^**	**Mouse**	(13)
Rv3804c	61–68	YDQSGLSV	**K^d^**	**Mouse**	(18)
Rv3804c/Rv1886c	71–78	PVGGQSSF	**L^d^**	**Mouse**	(18)
Rv3804c/Rv1886c	145–152	YAGAMSGL	**K^d^**	**Mouse**	(18)
Rv3804c	161–168	PTLIGLAM	**L^d^**	**Mouse**	(18)
Rv1886c	145–152	F**I**YAG**SL**S	HLA-A*0201	**HLA-tg**	(27)
Rv1886c	199–207	KL**V**ANNTR**L**	HLA-A*0201	**HLA-tg**	(27)
**PROTEIN VACCINATION (*Mtb* Ag85 COMPLEX)**					
Rv1886c	11–30	LQVPSPSMGRDIKVQFQSGG	HLA-DRA/B1*0302	**HLA-tg**	(27)
Rv3804c	121–145	AVVGL 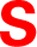 MAASSALTL	**Epimer**	**Guinea pigs**	(11)
Rv3804c	196–215	NVGKLIANNTRVWVYCGNGK	**Epimer**	**Guinea pigs**	(11)
Rv1886c	101–122	LTSELP**Q**WL**S**ANR**A**VKPTGSAA	**Epimer**	**Guinea pigs**	(11)
Rv1886c	126–140	SMAGSSA**M**ILA**A**YHP	**Epimer**	**Guinea pigs**	(11)
Rv1886c	261–275	T 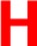 SWEYWGAQLNAMK	**Epimer**	**Guinea pigs**	(11)
***M. leprae* INFECTION**					
Rv3804c	11–30	LQVPSPSMGR DIKVQFQSGG	**Promiscuous**	**Lepromin+**	(23)
***M. ulcerans* PLASMID VACCINATION**					
MUL4987	21–40	**N**IKVQFQSGG ANSPALYLLD	**H-2^b^**	**Mouse**	(39)
MUL4987	61–80	Y**Y**QSG**I**SV**A**MPVGGQSSFYS	**H-2^b^**	**Mouse**	(39)
MUL4987	81–100	DWY**N**PACGKAGC **T**TYKWETF	**H-2^b^**	**Mouse**	(39)
MUL4987	240–259	**FQ**A**AYNA****A**G**GHNAVWNF**D**D**	**H-2^b^**	**Mouse**	(39)
MUL4987	261–280	T 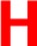 SWEYWGAQ LNAMRPDLQ**H**	**H-2^b^**	**Mouse**	(39)
***Map* ATCC 19698 INFECTION**					
Rv3804c	241–260	QDAYNAGGGHNGVFDFPDSG	**I-A^b^**	**B6 ^bg/bg^**	(41)
Rv1886c	241–260	QDAYNA**A**GGHN**A**VF**N**FPP**N**G	**I-A^b^**	**B6 ^bg/bg^**	(41)
Rv3804c	261–280	T 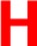 SWEYWGAQLNAMKPDLQR	**I-A^b^**	**B6 ^bg/bg^**	(41)
Rv1886c	262–279	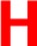 SWEYWGAQ LNAMK**G**D**L**Q	**I-A^b^**	**B6 ^bg/bg^**	(41)
Rv0129c	261–280	T 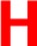 SW**P**YW**NE**Q L**V**AMK**A**D**I**Q**H**	**I-A^b^**	**B6 ^bg/bg^**	(41)
Rv0129c	21–40	DIKVQFQ**G**GG **PH**.A**V**YLLD	**I-A**^b^	**B6 ^bg/bg^**	(41)
Rv1886c	145–162	YAG**SL**S**A**LLDPSQ**G**MGPS	**Promiscuous**	***Bos taurus***	(41)
***Map* PLASMID DNA VACCINATION**					
Rv3804c	241–260	QDAYNAGGGH NGVFDFPDSG	**I-A^b^**	**B6**	(42)
Rv1886c	240–260	**F**QDAYNA**A**GGHN**A**VF**N**FPP**N**G	**I-A^b^**	**B6**	(42)
Rv3804c	91–110	GCQTYKWETF LTSELPGWLQ	**I-A^b^**	**B6**	(42)
Rv1886c	145–162	YAG**SL**S**A**LLDPSQ**G**MGP**S**	**I-A^b^**	**B6**	(42)

*Immunodominant T-cell epitopes of Ag85, as defined in experimental vaccination models and infection (LTBI, latent TB infection)*.

*Amino acids different from aa sequence of *Mtb*Ag85A (Rv3804c) are indicated in bold/underlined*.

*The three aa involved in mycolyl-transferase activity are highlighted in red*.

##### *M. tuberculosis* H37Rv

BALB/c and C57BL/6 mice were infected intravenously with *Mycobacterium tuberculosis* H37Rv and Th1-type spleen cell cytokine secretion was analyzed in response to purified Ag85A, Ag85B, and Ag85C components and synthetic overlapping peptides covering the three mature sequences (13). Tuberculosis-infected C57BL/6 mice reacted strongly to some peptides from Ag85A and Ag85B but not from Ag85C and more specifically strong responses were detected against peptide 25 (aa 241–260) of Ag85A and against the same sequence of Ag85B (13). This latter peptide region was also identified by Yanagisawa et al. (14) and TCR-transgenic mice with MHC class II A^b^-restricted CD4^+^ T-cells expressing TCR α and β chains for the mycobacterial **Ag85B_240–254_** have been generated (15). Tuberculosis-infected BALB/c mice reacted only to peptides from Ag85A: p11 (aa 101–120), p16 (aa 151–160), and p20 (aa 191–210) (13).

##### Plasmid DNA vaccines/viral vectors encoding Ag85A, Ag85B, and Ag85C of *Mtb*

Plasmid DNA vaccination is a powerful tool to identify protective antigens of tuberculosis and to identify immunodominant CD4^+^ and particularly CD8^+^ T-cell epitopes (16). BALB/c and C57BL/6 mice were vaccinated intramuscularly with plasmid DNA encoding the three components of the Ag85 complex. Ag85A and Ag85B encoding plasmids induced a robust Th1 like response to native Ag85 purified from BCG CF, characterized by elevated levels of IL-2, IFN-γ, and TNF-α. Levels of IL-4, IL-6, and IL-10 were low or undetectable. Plasmid encoding Ag85C was only weakly immunogenic. Whereas BALB/c mice reacted preferentially to the Ag85A component, C57BL/6 mice reacted to both Ag85A and Ag85B with more or less the same magnitude (17). Furthermore, vaccination with plasmid DNA encoding Ag85A or Ag85B but not Ag85C conferred significant protection against mycobacterial replication in lungs from C57BL/6 mice (17). T-cell epitopes could be identified in BALB/c and C57BL/6 mice vaccinated with plasmid DNA encoding Ag85A, Ag85B, and Ag85C DNA using synthetic peptides spanning the three Ag85 proteins, and the epitope repertoire was found to be broader than in infected mice (13). Despite pronounced sequence homology, a number of immunodominant regions contained component specific epitopes. Thus, BALB/c mice vaccinated against all three Ag85 antigens reacted against the same amino-acid region, **101–120** (already identified in BCG vaccinated and TB infected mice) but responses were completely component specific. In C57BL/6 mice, a cross-reactive T-cell response was detected against two carboxy-terminal peptides spanning amino acids 241–260 and 261–280 of Ag85A and Ag85B. These regions were not recognized at all in C57BL/6 mice vaccinated with Ag85C DNA.

**Figure 2:**
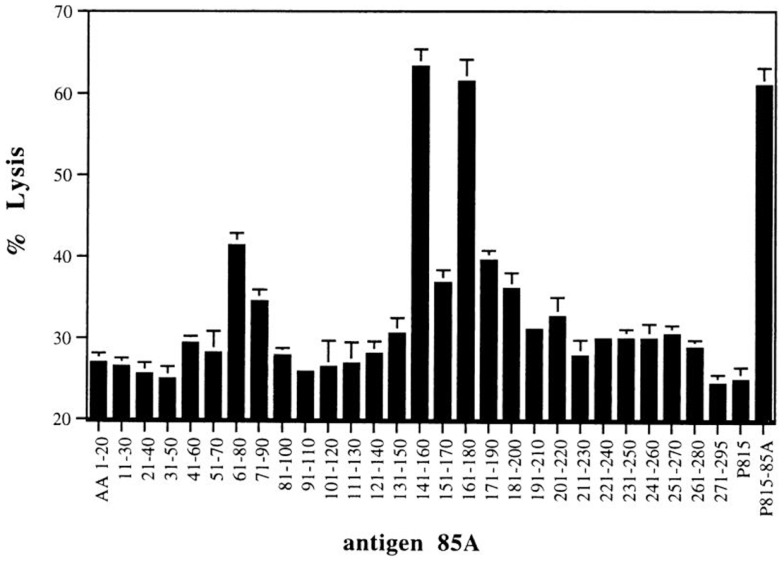
**CTL activity against P815 target cells loaded with synthetic 20-mer peptides (overlapping by 10 aa, covering the complete Ag85A sequence) of spleen cells from BALB/c mice vaccinated with mature Ag85A DNA**. Reproduced from Ref. (18).

T-cell repertoire of BALB/c mice vaccinated with plasmid DNA encoding Ag85A was broader that of *Mtb* infected mice (13, 18). Besides peptide regions spanning aa **11–30** and **191–210** inducing both IL-2 and IFN-γ responses, three peptides induced strong IFN-γ but weak to no IL-2 responses. More detailed analysis of the Ag85A sequence for predicted MHC class I binding motifs using “human leukocyte antigen (HLA) peptide motif” (http://www-bimas.cit.nih.gov/molbio/hla_bind/) showed that these three peptides spanned four predicted CD8^+^ T-cell epitopes (18). The following half-time dissociation scores (reflecting affinity for the respective MHC class I molecules) were found: aa **61–68** YDQSGLSV: half-time dissociation score 600, predicted K^d^; aa **71–78** PVGGQSSF: half-time dissociation score 390, predicted L^d^; aa **145–152** YAGAMSGL: half-time dissociation score 2000, predicted K^d^; aa **161–168** PTLIGLAM: half-time dissociation score 150, predicted L^d^. CTL activity against these peptides was demonstrated using a ^51^Cr release assay (Figure 2) and showed cross-reactive responses against Ag85B for both K^d^ restricted peptides (18). A particularly interesting region was identified in peptide 15 spanning aa 141–160, which besides the K^d^ restricted epitope 145–152 also contains a CD4^+^ epitope with a predicted Rothbard and Taylor motif spanning aa 147–154 and an amphipathic stretch spanning aa 149–157 (according to T sites program) (19).

Immunization with DNA followed by modified vaccinia virus Ankara strain, both expressing the antigen 85A, induced both CD4^+^- and CD8^+^-T-cell responses in BALB/c mice, directed against the K^d^ restricted epitope WYDQSGLSV (aa 60–67) and the I-E^d^ restricted epitope TFLTSELPGWLQANRHVKPT (aa 99–119), respectively (20). DNA priming, followed by a MVA85A boost induced both CD4^+^ and CD8^+^ responses, whereas priming with MVA followed by a DNA boost only induced CD4 responses. Following immunization with dendritic cells pulsed with the antigen 85A CD4^+^- or CD8^+^-restricted epitope, alone or in combination, copresentation of both epitopes on the same dendritic cell was required for protection, demonstrating that induced CD8^+^ T-cells can play a protective role against tuberculosis (20).

A single intranasal, but not i.m., immunization with a recombinant replication-deficient adenoviral-based vaccine expressing Ag85A (AdAg85A) provided potent protection against airway *M. tuberculosis* challenge at an improved level over that by cutaneous BCG vaccination. Systemic priming with an Ag85A DNA vaccine and mucosal boosting with AdAg85A conferred a further enhanced immune protection, which was remarkably better than BCG vaccination. Such superior protection triggered by AdAg85 mucosal immunization was correlated with much greater retention of Ag-specific T-cells, particularly CD4 T-cells, in the lung and was shown to be mediated by both CD4+ (LTSELPGWLQANRHVKPTGS, aa 101–120) and CD8^+^ (MPVGGQSSF, aa 70–78) T-cells (21).

For H-2^b^ haplotype mice, no MHC class I restricted epitopes have been identified so far on Ag85A or Ag85B to our knowledge, neither in BCG or plasmid DNA vaccinated nor in TB infected mice.

#### Human studies

##### Tuberculosis

In our first paper on Ag85A (called P32 at that time), we reported that healthy Mantoux positive volunteers showed a much stronger lymphoproliferative and IFN-γ response to this antigen than tuberculosis patients (22). This was the initial indication that T-cell responses against this protein could confer protection against *Mtb*. Subsequently, we reported on T-cell epitope mapping of Ag85A from *Mtb* using peripheral blood mononuclear cell (PBMC) cultures from healthy tuberculin-positive volunteers and from patients with tuberculosis, using the same synthetic 20-mer peptides of the murine study. Peptide recognition was largely promiscuous, with a variety of HLA haplotypes reacting to the same peptides. PBMC from all tuberculin-positive subjects reacted to Ag85A, and the majority proliferated in response to peptide 6 (amino acids 51–70), peptides 13, 14, and 15 (amino acids 121–160), or peptides 20 and 21 (amino acids 191–220). PBMC from tuberculosis patients demonstrated a variable reactivity to Ag85 and its peptides, and the strongest proliferation was observed against peptide 7 (amino acids 61–80) (23). Nine out of ten of the tuberculin-positive volunteers in this study reacted to aa **141–160**, precisely the peptide characterized by the presence of both a CD4^+^ and a CD8^+^ epitope in BALB/c mice. In contrast, the most immunogenic CD4^+^ peptide of Ag85A for BALB/c mice, i.e., p11 was not recognized by PBMCs from healthy PPD-positive humans. However, two reports published in 2000 and 2001 showed that aa **100–117** of **Ag85B** is recognized in a similar promiscuous manner by T-cells from a majority of PPD-positive human volunteers (24, 25). Ag85A differs from Ag85B in three aa in this region Gly107Gln, Gln110Ser, and His114Ala. Ag85A sequences from *M. ulcerans, M. leprae, Map*, and *Maa* also show strong differences as compared to *Mtb*Ag85A sequence in this region (see Figure 1). These aa shifts probably explain the difference in the human responses to Ag85A and Ag85B, as also in DNA vaccinated BALB/c mice, IL-2 and IFN-γ responses to region 100–120 are specific for both Ag85 components (13).

In 1995, Silver et al. had already assessed the T-cell epitopes of *Mtb* Ag85B using blastogenic responses of PBMC from 12 healthy purified protein derivative-positive subjects to a set of synthetic 15-mer peptides based on the full 325-amino-acid sequence (leader sequence included) (26). Seven immunodominant regions were identified and each subject responded to at least one of the two most dominant epitopes, which corresponded to aa **91–115** and aa **193–217**. Peptides of these two epitopes induced production of IFN-γ by sorted CD4^+^ T-cells.

Human leukocyte antigen-transgenic mice can be a powerful tool to identify human T-cell epitopes in an experimental mouse model. In 1998, A. Geluk reported on the identification of an HLA-class II restricted epitope of Ag85. HLA-DRA/B1*0302 (DR3) transgenic mice were vaccinated with Ag85 protein, purified from *Mtb* CF in Incomplete Freund’s adjuvant (27). Using 20-mer peptides, covering the entire *Mtb* 85B sequence, they identified one single peptide epitope in the NH_2_-terminal region, spanning aa **11–30** (aa 51–70 in the numbering including the signal peptide): LQVPSPSMGRDIKVQFSGG. This sequence is identical in Ag85A and Ag85B, but differs in two positions in Ag85C: Pro16Ala and Ser28Gly. Also in *M. ulcerans, Map*, and *Maa*, position 16 has the shift to Alanine. In *M. leprae* there is also one aa shift: Ser28Asn.

Whereas most of these studies on human T-cell epitope mapping were performed during the mid-nineties, one more recent paper of Lindestam Arlehamn et al. reported on the memory phenotype of *Mtb*-specific CD4^+^ T-cells, using HLA-class II tetramers for a peptide shared between Ag85A and Ag85B, i.e., aa **90–104** AGCQTYKWETFLTSE in healthy PPD-positive donors, latently infected with *Mtb* (28). Tetramer positive T-cells predominantly consisted of CD45RA^−^CCR7^+^ central memory T-cells in all donors tested, followed by effector memory (CD45^−^CCR7^−^) T-cells. Only a minor fraction appeared to be naïve or effector T-cells. Interesting to note that this sequence is shared with Ag85A of *M. leprae* that there is only Q93T shift in *M. ulcerans, Map*, and *Maa* but that the sequence of *Mtb* Ag85C differs in five positions.

Less is known on human MHC class I restricted epitopes of Ag85. Klein et al. reported on a HLA-B*35 restricted CD8^+^ T-cell epitope of Ag85C (29). Using reverse immunogenetics, they tested 23 motif-bearing peptides of the Ag85 complex for binding to HLA-B*35, one of the most common HLA-B types in West Africa. Three 9-mer peptides bound with high affinity to HLA-B*3501. Peptide MPVGGQSSFY (spanning aa 70–79 of the mature protein), a highly conserved region shared by all three members of the Ag85 complex of *Mtb* and also identical in *M. ulcerans, M. avium*, and *M. leprae*. This peptide encompasses an L^d^ predicted epitope, recognized by BALB/c mice vaccinated with pAg85A DNA (see Plasmid DNA vaccines/viral vectors encoding Ag85A, Ag85B, and Ag85C of *Mtb*) and also an IL-2/IFN-γ inducing region for C57BL/6 mice vaccinated with pAg85C (13). Peptide WPTLIGLAM of Ag85C (spanning aa 160–168) with a W160G change as compared to all other sequences, and a T162S change in Ag85B, and the three other non-tuberculous mycobacteria. Finally peptide IPAEFLENF of Ag85B (spanning aa 224–232), with an isoleucine in position 224 shared with Ag85C, and a leucine in Ag85A of *Mtb* and the four non-tuberculous mycobacteria. WPTLIGLAM stimulated effector cells were able to kill *Mtb* or BCG infected macrophages and produced IFN-γ and TNF-α (30). Interestingly, an L^d^ restricted epitope spanning the same aa 161–168 (PTLIGLAM) was identified in Ag85A DNA vaccinated BALB/c mice, which did not cross-react with the corresponding Ag85B peptide (because of the Thr162Ser shift).

A comprehensive epitope mapping to HLA-A*0101, A*0201, A*1101, A*2402, B*0702, B*0801, and B*1501 of Ag85B was published in 2007 (31). Affinity and half-life (t_1/2_ off-rate) analysis for individual peptide species on HLA-A and HLA-B molecules revealed binding ranges between 10^−3^ and 10^−7^ M. After selection of the best matches, major histocompatibility complex class I/peptide tetramer complexes were constructed to measure the CD8^+^ T-cell responses directly *ex vivo* in PBMC derived from 57 patients with acute pulmonary tuberculosis. Three patterns of (allele-) specific CD8^+^ recognition were identified: (a) Focus on one dominant epitope, (b) Co-dominant recognition of two distinct groups of peptides, and (c) Diverse and broad recognition of peptides (presented by HLA-A*0201). Peptides that bound with slow off-rates to class I alleles, that is HLA-A*0201, were associated with low frequency of CD8^+^ T-cells in PBMCs from patients with tuberculosis. HLA-B alleles showed fast off-rates in peptide binding and restricted high numbers (up to 6%) of antigen-specific CD8^+^ T-cells in patients with pulmonary tuberculosis (31). Functional analysis (*in vitro* IL-2 and IFN-γ production) revealed that tetramer-binding T-cells in PBMCs from these patients were little or not responsive to the nominal peptide epitope, confirming the notion of a deficient Ag85 specific T-cell response in TB patients. The study focused on TB patients and not on latently infected subjects, which could have been more relevant in the context of TB vaccine development.

##### *M. bovis* BCG

In 1994, Roche et al. reported on the T-cell determinants of Ag85B of *M. bovis* (MPB 59) in BCG vaccinees and TB patients. The mature 85B protein of *M. bovis* (MPB59) has a high degree of amino-acid identity with the *M. bovis* 85A protein (76%) and the *Mtb* 85B (99%) and *Mtb*85A (76%) proteins. Proliferative assays with recombinant MPB59 demonstrated that PBMC from 95% of BCG vaccinees and 52% of tuberculosis patients responded to the whole mature protein. Using a set of synthetic 20-mer peptides, five peptides were found to be recognized in more than half of the MPB59 responders. The T-cell-reactive regions were essentially identical in the *M. bovis* and *Mtb* 85B proteins. Subjects with a variety of HLA-DR phenotypes responded to a number of these peptides and there was no difference in the pattern of responses between BCG vaccinees and TB patients. A promiscuous recognition pattern was observed in response to peptides spanning aa 51–70 (recognized by 87% of the responsive BCG vaccinees and 93% of the responding TB patients) and aa 11–30 (recognized by 73% of the responders). Peptides in the C-terminal region (aa 131–150 and aa 191–210) were more frequently recognized by patients than by BCG vaccinees (32). More recently, Finan et al. reported on 236 healthy Gambian babies vaccinated at birth with *M. bovis* BCG (33). Using a whole blood assay 2 months after vaccination, cytokine analysis showed that 89% of the babies produced Ag85 complex specific IFN-γ responses, albeit that response varied up to 10 log-fold within this population and 25 and 31% of the babies also produced detectable levels of the Th2 cytokines IL-5 and IL-13, respectively. Unfortunately, T-cell epitopes were not mapped in this study.

A. Geluk et al. reported on the identification of two HLA-A*0201 restricted CD8^+^ T-cell epitopes of Ag85B using pDNA vaccination encoding Ag85B of HLA-A2/K^b^ transgenic mice. (34). HLA-A*0201 is one of the most prevalent class I alleles, with a frequency of over 30% in most populations. The two peptides spanned aa 145–152 FIYAGSLS and aa 199–207 KLVANNTRL. As already mentioned, the first region is also recognized by K^d^ restricted CD8^+^ T-cells of Ag85A/B DNA vaccinated mice. The second peptide differs from that of Ag85A only in position 201, with a leucine in the Ag85A and a valine in the Ag85B sequence (both with a non-polar side chain), change that does not affect the binding affinity for HLA-A*0201 (34). As precursor frequencies of these cells were low in the periphery of human BCG vaccinees, restimulation with *M. bovis* BCG was necessary to visualize the cells by tetramer staining. Stable human CD8^+^ T-cell lines were generated against the two peptides, using CD4 depletion and peptide-pulsed autologous Dcs derived, from HLA-A*0201+ BCG-responsive donors. These T-cell lines were able to lyse HLA-A*0201+ peptide-pulsed targets and produced the pro-inflammatory cytokines IFN-γ and TNF-α. The group of H. Dockrell also demonstrated Ag85A specific CTL responses using BCG-specific cell lines generated from PBMC of BCG-vaccinated donors stimulated for 2 weeks with live *M. bovis* BCG in the presence of IL-2 and IL-7 (35). In this study, two HLA-A*0201 restricted epitopes were identified, one spanning aa 5–13 GLPVEYLQV and the other spanning aa 199–207 KLIANNTRV. This second peptide spans exactly the same region of Ag85B identified by Geluk et al., using HLA-A2/K^b^ transgenic mice, suggesting the existence of a cross-reactive CTL epitope in this region for Ag85A and Ag85B.

##### Leprosy

In 1994, we also reported on T-cell epitope mapping of *Mtb*Ag85A using PBMCs from healthy lepromin-positive volunteers and from patients with leprosy. As for tuberculosis, peptide recognition was largely promiscuous, with a variety of HLA haplotypes reacting to the same peptides. However, despite a 90% homology between the 85A proteins of *M. leprae* and *Mtb*, the peptides recognized were different. PBMC from lepromin-positive healthy contacts reacted against peptide 2 (aa 11–30), peptide 5 (aa 41–60), and peptides 25 and 26 (aa 241–270). PBMC from paucibacillary patients reacted preferentially against peptide 1 (amino acids 1–20) and peptide 5. Multibacillary patients were not reactive to Ag85 or the Ag85A peptides (23). It is interesting to note that responses to aa 11–30 were also identified in BCG vaccinees (27, 32). It is well known that BCG vaccination exerts some degree of protection against leprosy and that a second BCG immunization can increase this protection (36).

#### Guinea pigs

Lee and Horwitz reported on T-cell epitope mapping of Ag85A and Ag85B in outbred Hartley strain guinea pigs, immunized with the purified *Mtb* proteins and tested for splenocyte proliferation in response to a series of overlapping 15-mer peptides spanning the mature proteins (11). Three of the nine immunoreactive regions of Ag85B identified in the guinea pigs (aa 101–122, 126–140, and 261–275) overlapped with epitopes predicted by the EpiMer computer program (37). Two immunodominant T-cell epitopes were identified in Ag85A immunized guinea pigs, spanning aa 121–145 and 196–215, and these regions were also predicted by the EpiMer program (37).

### T-cell epitopes of Ag85 of *M. ulcerans*

The gene encoding Ag85A from *M. ulcerans* 5150 (MUL4987) shares 84.1% amino-acid sequence identity and 91% conserved residues with the gene encoding Ag85A from *Mtb* (38) (see also sequence alignment). We characterized the H-2^b^ restricted immunodominant T-cell epitopes, using synthetic 20-mer peptides spanning the entire mature sequence of Ag85A from *M. ulcerans* and from *Mtb* (39). *M. ulcerans* DNA vaccinated mice reacted against *M. ulcerans* peptides both from the NH_2_-terminal and COOH-terminal part of the protein, whereas *Mtb* DNA vaccinated mice reacted almost exclusively against *M. ulcerans* peptide spanning aa 240–259, albeit that its sequence is quite different from that of *Mtb*. *M. ulcerans* DNA vaccinated mice also recognized this peptide very effectively. Responses against the NH_2_-terminal peptides spanning aa 61–80 and 81–100 of *M. ulcerans* were only observed in *M. ulcerans* DNA vaccinated mice, indicating that this NH_2_-terminal region was responsible for a partial species-specificity.

### T-cell epitopes of Ag85 of *M. avium* subsp. *paratuberculosis (Map)*

The genes encoding the three Ag85 components from *M. avium* subsp. *paratuberculosis* (*Map)* have been sequenced, and at the protein level, a 99% sequence identity with *M. avium* subsp. *avium (Maa)* was found, with a single amino-acid residue difference for each protein: Ag85A: Ser155Pro, Ag85B: Ser120Asn, and Ag85C: Ileu284Thr for *Map* vs. *Maa*. Compared to the mature protein sequences of *M. bovis, Map*85A (Map 0216) shares 82%, *Map*85B (Map 1609c) shares 86%, and the *Map*85C (Map 3531c) shares 87% identity (40). *Map* ATCC 19698 was adapted to grow as a surface pellicle on synthetic, protein-free Sauton medium supplemented with mycobactin J. Comparison of the 4-week-old *Map* CF with the protein profile of a 2-week-old *Mtb* H37Rv CF by SDS-PAGE indicated that in the region of the Ag85 complex, only one protein of approximately 30 kDa (presumably Map1609c) was strongly expressed in *Map* CF (41).

**Figure 3:**
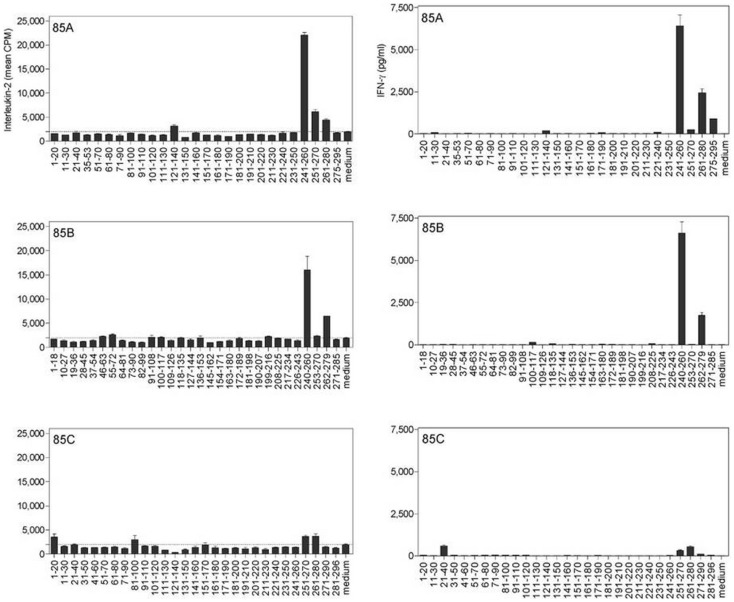
**Characterization of cross-reactive T-cell epitopes of Ag85A, Ag85B and Ag85C in *M. avium* subsp*. paratuberculosis*-infected B6^bg/bg^ mice, using synthetic peptides spanning the mature sequences of Ag85A, Ag85B, and Ag85C from *Mtb***. IL-2 (left) and IFN-γ (right) production was analyzed in 24 and 72-h culture supernatants, respectively, of spleen cells from a pool of five animals infected intravenously with 3 × 10^5^ CFU of *Map* and stimulated with overlapping synthetic peptides (10 μg/ml). Reproduced from Ref. (41).

Cross-reactive CD4^+^ epitopes of Ag85A, Ag85B, and Ag85C of *Mtb* were identified in H-2^b^ mice intravenously infected with *Map* ATCC 19698 (Figure 3) (41). Spleen cells from susceptible *Map* infected B6^bg/bg^ mice reacted against peptides of Ag85A and Ag85B from *Mtb*. These epitopes were the same as those we have previously identified in B6 mice infected with *Mtb* or vaccinated with DNA encoding the *Mtb* Ag85 components (13). Peptides from Ag85C were more weakly recognized by spleen cells from *Map* infected mice.

Cross-reactive CD4^+^ epitopes of Ag85A, Ag85B, and Ag85C were also identified in C57BL/6 mice vaccinated with plasmid DNA encoding the *Map* antigens (42). Plasmid DNA encoding the *Map*Ag85A component induced the strongest IFN-γ response, whereas DNA encoding *Map*Ag85B and *Map*Ag85C induced about 10-fold lower titers. More or less the same epitopes were recognized by pDNA vaccinated as by *Map* infected mice. However, and in contrast to infection, peptide 27 of Ag85A or peptide 27 from Ag85C were not recognized in mice vaccinated with the respective DNAs. On the other hand, three new epitopes were identified in pDNA vaccinated mice that were not recognized in infected animals: peptide 10 (aa 91–110) on Ag85A, peptide 17 (aa 145–162) on Ag85B, and the corresponding region of Ag85C covered by peptides 15–16 (aa 141–170). The strongest Th1 epitope identified in this study spanned region 241–260 of the Ag85A and Ag85B sequence. These sequences are very similar to each other, but – as for *M. tuberculosis –* the Ag85C sequence is completely different.

Ag85 specific T-cell epitopes were also mapped in experimentally infected cattle, the target species of Johne’s disease. Five 2- to 3-week-old calves were infected by the oral route with 10 mg (10^8^ CFU) of *Map* (ATCC 19698) cells per day for 10 consecutive days. (41). Strong proliferative and *ex vivo* IFN-γ responses against Ag85, purified from *M. bovis* BCG CF, could be detected in cattle as early as 10 weeks after oral *Map* infection. Synthetic peptides from the Ag85A and Ag85B components of this complex were strongly recognized, whereas T-cell responses were weaker against peptides from the Ag85C protein. A promiscuous T-cell epitope spanning amino acids 145–162 of Ag85B (identical sequence in *M. bovis* and *Map*) was identified in experimentally infected cattle.

## Concluding Remarks

Members of the Ag85 complex are immunodominant mycobacterial antigens, which induce strong Th1-type immune responses in situations of controlled mycobacterial infection. By virtue of their role in cell wall integrity and synthesis of cord factor, these abundantly expressed proteins have long been considered as virulence factors. On the other hand, because of the strong T-cell responses they induce, these proteins can also be regarded as a means of both host and pathogen to reach a state of equilibrium, advantageous for both parties. An estimated one third of the world population is infected with *Mtb*, an immense reservoir for this successful microbe. However, most humans latently infected with *Mtb* will never develop the active disease, precisely because of the strong elicited T-cell response. A similar scenario holds true for infections caused by other mycobacteria such as *M. leprae* and non-tuberculous mycobacteria from the environment such as *M. ulcerans* and various *M. avium* subspecies. The MHC class I and MHC class II restricted epitopes of *Mtb*/*M. bovis* Ag85A and Ag85B have been identified in experimental animal models and in healthy Mantoux positive subjects. In humans, a small number of dominant T-cell epitopes were found to be promiscuously recognized by subjects with many different HLA haplotypes. Also in experimental mouse models (particularly of H-2^d^ haplotype), the same epitopes have been identified. More in particular, regions spanning aa 10–30, 60–80, 100–120, 140–160, and 199–207 of the mature Ag85A and Ag85B span these IFN-γ/IL-2 inducing Th1/CTL epitopes. The Ag85C component differs in its sequence from the two other components and overall T-cell responses against this third component are lower. The Ag85C molecules are buried more in the cell wall and hence may be less accessible for rapid antigenic processing and presentation to T-cells. It is also possible that because of this less exposed localization, there has been less evolutionary pressure on the gene of Ag85C to encode for such immunodominant, promiscuous T-cell epitopes. Members of the Ag85 complex are highly conserved in other mycobacterial species and cross-reactive T-cell responses against *Mtb* antigens can be found in *M. ulcerans, Map*, and *M. leprae* infection. Sequence comparisons indicate that the Ag85A sequences of these three non-tuberculous mycobacteria are more similar to the sequence of *Mtb*Ag85B than of *Mtb*Ag85A, particularly in aa stretches spanning the immunodominant epitope regions. Moreover, expression of the two components by different mycobacterial species seems to be differentially regulated, with a preference for the Ag85B orthologs in NTM.

Recently, a randomized, placebo-controlled phase 2b trial in a rural region near Cape Town, South Africa, analyzing safety and efficacy of MVA85A in infants previously vaccinated with BCG, showed that the MVA85A boost was well tolerated but induced only modest cell-mediated immune responses (lower that responses observed in previous studies in adult BCG vaccinees in United Kingdom) and furthermore did not augment protective efficacy of BCG (43). The reasons for this vaccine trial failure are not clear but conclusions as to the protective nature of Ag85A should not be taken too hastily. It is important to stress that the rationale for all BCG boosting strategies is based on the assumption that BCG-induced protection is waning in time through gradual attrition of BCG-induced T-cells. One could argue that in the Tameris study, the time between neonatal BCG vaccination and MVA85A boost was too short to measure effects of waning immunity. On the other hand, there may be other factors than just waning that are responsible for the variable efficacy of BCG and besides magnitude, the *quality* of the memory response induced by the BCG vaccine may be insufficient (44). More specifically, the BCG vaccine is a very poor inducer of CD8^+^ T-cells, which are especially important for the control of a latent TB infection. As BCG vaccination primes almost exclusively for MHC class II restricted responses, it is obvious that boosting strategies with proteins and even with recombinant viral vectors will augment preferentially the CD4^+^ T-cell population. Priming with plasmid DNA encoding Ag85A can increase the protective efficacy of BCG in mice as measured in a long term survival study and this increased efficacy is accompanied by increased Ag85A specific CD8^+^ responses (45). More recently, we have shown in an experimental mouse model and also in a large mammalian species (*Sus scrofa*) that the vaccine potential of live BCG can be augmented by coadministration with plasmid DNA encoding PPE44 and Ag85A respectively, as measured by Th1-type cytokine secretion, specific IgG antibodies, as well as specific IFN-γ producing CD8^+^ T-cells (46) (Bruffaerts et al. submitted for publication). These results have provided a proof of concept for a new TB vaccine, based on BCG-plasmid DNA combination, approach that now needs to be tested in non-human primates, the only animal species in which reactivation of latent *Mtb* infection can be monitored properly (47).

## Conflict of Interest Statement

The author declares that the research was conducted in the absence of any commercial or financial relationships that could be construed as a potential conflict of interest.
